# Descriptive norms can “backfire” in hyper-polarized contexts

**DOI:** 10.1093/pnasnexus/pgae303

**Published:** 2024-10-15

**Authors:** David G Rand, Erez Yoeli

**Affiliations:** Sloan School of Management, Massachusetts Institute of Technology, 100 Main Street, Cambridge, MA 02142, USA; Sloan School of Management, Massachusetts Institute of Technology, 100 Main Street, Cambridge, MA 02142, USA

**Keywords:** social norms, descriptive norms, polarization, public health messaging

## Abstract

Descriptive social norms interventions, where a behavior is promoted by learning that others engage in that behavior, are a cornerstone of behavior change research and practice. Here, we examine the effect of learning about the behavior of outgroup members in a hyper-polarized context: mask-wearing during the COVID-19 pandemic. Contrary to prior findings, we find a descriptive social norm “backfire”: Across three experiments, Biden supporters increased their mask-wearing intentions after being informed that most Trump supporters never wore masks. We also provide evidence consistent with a mechanism whereby this effect was driven by changes in perceptions about how negatively ingroup members view nonmask wearing. Finally, in a fourth study, Biden supporters show the traditional descriptive norms effect (rather than a backfire) from the same treatment when in a nonpolarized context: dishonesty in a coin-flipping task. These findings help to clarify why descriptive social norm interventions promote behavior change, and underscore the importance of social norms in motivating prosocial behaviors. They also suggest an update to current best practices in the design of descriptive norm interventions: in polarized contexts, it can be beneficial to publicize antisocial behavior of outgroup members.

**Significance Statement**

A “descriptive social norm” intervention provides information about a prevailing behavior. This has been shown to motivate people to change their behavior to comply with the norm. However, what if the descriptive social norm is used in a polarized context, and describes the behavior of people whose beliefs and behaviors oppose one’s own? In such a case, people may change their behavior in the opposite way, leading to a “backfire” effect. To illustrate this, we tell Biden supporters that most Trump supporters refuse to wear masks to help abate the spread of COVID-19. The Biden supporters respond with stronger intentions to wear masks. However, when we show Biden supporters information about whether Trump supporters lie in a “coin flip” task—a setting that is not polarized—we find no backfire effect. Our findings help to clarify why normative information motivates behavior change, to clarify how to best use normative information in polarized environments, and also to alleviate concerns that widespread publicity of anti-maskers reduced mask-wearing by the average American.

## Introduction

In recent years, descriptive social norms have emerged as one of the most widely used “nudges” in behavioral science. A wealth of studies have shown that informing people that others are engaging in a given behavior increases people’s own likelihood of engaging in that behavior (1). This approach has been used to successfully change behavior across a wide range of contexts, including water conservation (2, 3), energy conservation (4–6), cheating (7), tax compliance (8), compliance with workplace guidelines (9), mask-wearing intentions during the COVID-19 pandemic (10–12), the resumption of economic activity after COVID-19 lockdowns (13), bullying at school (14), and counternormative sustainability behaviors via dynamic norms (15). It has even been used to alter people’s judgments of “right” and “wrong” (16).

A canonical observation regarding descriptive norms is that the more dissimilar the reference group is to the participant, the less of an effect the descriptive norm information has on behavior. For example, in a classic study attempting to increase towel reuse in a hotel, Goldstein et al. (2) found that information about the behavior of others was more effective when this information was about guests of the same gender, or even those who stayed on the same floor, and in the same room. Similarly, Vives et al. (17) found that learning about ingroup members behaving immorally increased immoral action, whereas learning about outgroup members behaving immorally had no effect. From a theoretical perspective, the behavior of dissimilar others provides less information about how you are expected to behave by those you interact with—that is, the behavior of dissimilar others tells you less about the social norms you are bound by (2, 18–21).

Here, we shed new light on descriptive social norms interventions by examining an even more extreme situation: What happens when you learn about the actions of a highly salient outgroup in the context of a highly polarized behavior? Specifically, we test the effect of informing Biden supporters that most Trump supporters are choosing not to wear masks to prevent the spread of COVID-19. The COVID-19 pandemic in general, and masking in particular, became extremely polarized in the US (22–24)—during the height of the pandemic, most Democrats strongly favored mask wearing while many Republicans strongly opposed it. As such, mask-wearing became a major front in the cultural wars between left and right in the US. This created normative pressure for Democrats to mask, and for Republicans not to mask.

Given this context, we hypothesized that as opposed to the Biden supporters simply ignoring the Trump supporters’ behavior because they are seen as highly dissimilar, the descriptive norm could backfire, such that Biden supporters would shift their behavior away from the reported behavior of the Trump supporters. Theoretically, one reason for this is that descriptive norms can communicate information about the behaviors one is expected to comply with to avoid social sanctions (1, 2, 18, 20, 25, 26). In a highly polarized context like masking, the behavior of outgroup members is not simply irrelevant and thus to be ignored. Instead, ingroup and outgroup social norms about polarized behaviors are often diametrically opposed. Therefore, learning that the outgroup engages in a given behavior (not wearing masks) may communicate that the ingroup sanctions this behavior—leading to an increased perception of social pressure to reduce that behavior.

Consistent with this possibility, in Study 1 we find that informing Biden supporters that 9 out of 10 Trump supporters planned to never wear masks significantly increased Biden supporters’ mask-wearing intentions, whereas informing them that 9 out of 10 Biden supporters or Americans had no effect. Study 2 replicates the Trump norm backfire effect, and Study 3 provides evidence for the mechanism predicted by our theoretical account: learning that Trump supporters are not wearing masks causes participants to expect ingroup members (Biden supporters, and friends/family) to judge not wearing a mask more negatively. Finally, Study 4 shows that no such backfire occurs in the context of a nonpolarized behavior unrelated to political beliefs and affiliation: self-serving dishonesty on an incentivized coin-flipping task. Informing Biden supporters that many Trump supporters cheated on the task increased their own cheating.

## Study 1: descriptive social norms in a polarized context

### Participants

This study was conducted from 2022 May 3 to 2022 May 11, recruiting Americans using Prolific. Participants were restricted to Democrats who preferred Joe Biden to Donald Trump. We excluded Trump supporters from the masking studies because a pilot found that there was very little variation in their masking intentions (floor effect). Balancing statistical power with available budget, we aimed to recruit 1,500 participants for this experiment. In total, 1,659 participants (screened by Prolific to be Democrats) began the study; 69 were not allowed to complete the study because they reported preferring Donald Trump to Joe Biden; and 21 participants did not complete the key outcome measure. Thus, our final sample consisted of 1,583 participants (mean age = 35 years, age range = 18–82; 781 men, 774 women, and 28 who responded “Nonbinary/third gender”).

### Materials and procedure

The study began with a consent form, followed by a series of demographic questions, including “Who would you rather have as President?” (Joe Biden/Donald Trump). Participants who selected Donald Trump were told they were not eligible for the study.

After completing the demographic questions, participants were randomized into the control or one of three treatment conditions: the Trump treatment, the Biden treatment, or the Americans treatment. In the control, participants received the following instructions:

In a recent study, we asked people how often they planned to wear masks indoors. Over the next month, how often do you plan to wear a mask indoors? [Never, Infrequently, Occasionally, Frequently, Always]

In the Trump treatment, participants were also provided with descriptive social norm information indicating that most Trump supporters were not masking, immediately prior to being asked about their own masking intentions:

In a recent study, we asked people how often they planned to wear masks indoors. Out of 10 Trump supporters we asked, 9 said that they would never wear masks indoors. Over the next month, how often do you plan to wear a mask indoors? [Never, Infrequently, Occasionally, Frequently, Always]

The Biden treatment and Americans treatment were identical to the Trump treatment except that “Trump supporters” was replaced with “Biden supporters” or “Americans,” respectively.

These statistics reported to participants were in fact based on responses to an existing previous pilot study, but the 10 people were sampled in a nonrandom way in order to create the impression of a descriptive social norm against mask wearing.

They were then asked what fraction of Biden and Trump supporters they thought were going to wear masks indoors over the next month.

Finally, to mitigate the possibility that our study would reduce mask-wearing by participants, participants in the treatment conditions received a debriefing stating “Earlier in the study, we told you 9 out of 10 people from a recent study said they planned to never wear masks indoors. However, the overall result from the whole previous study was different from what those particular 10 people said. Overall, most people WERE still sometimes planning on wearing masks indoors. High-quality masks help keep you and others safe from COVID-19.”

All studies in this paper were deemed exempt by the MIT Committee on the Use of Human Subjects, protocol E-4389, and all participants provided informed consent at the study outset.

### Results

**Fig. 1.**
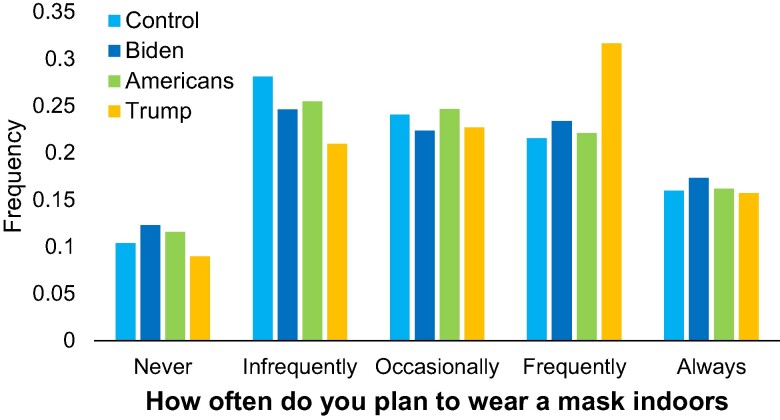
Distribution of masking intentions by condition in Study 1 (N=1,583). Control participants were given no information; participants in the treatment conditions were told that 9 out of 10 Biden supporters, Americans, or Trump supporters reported not planning to wear masks.

Mask wearing intentions by condition are shown in Fig. 1. Linear regression predicting mask wearing intentions using indicator dummy variables for the three treatments found that participants in the Trump treatment reported that they intended to wear masks significantly more frequently than participants in the control, d=0.16; t(1,579)=2.21, P=0.027, whereas the other two conditions had no significant effect (Biden treatment: d=0.03; t(1,579)=0.48, P=0.633; American treatment: d=0.01; t(1,579)=0.15, P=0.879). Furthermore, the effect of the Trump treatment was significantly larger than the effect of the American treatment, F(1,1579)=4.22, P=0.0402, and marginally significantly larger than the effect of the Biden treatment, F(1,1579)=3.03, P=0.0821).

Examining treatment effects on participants’ perceptions of the mask-wearing plans of others’, we found that the fraction of Biden supporters that participants expected to wear masks indoors was 49.2% in the control, and was significantly decreased relative to the control in the Biden treatment (6.9 pp, t=−3.20, P=0.001), did not significantly differ from the control in the Americans treatment (1.4 pp increase, t=0.64, P=0.52—perhaps because participants imagined most of those nonmask wearing Americans to be Trump supporters), and marginally significantly increased in the Trump treatment (3.7 pp, t=1.71, P=0.087). Conversely, expectations of the fraction of Trump supporters that participants expected to wear masks indoors was only 9.6% in the control, and—likely because of floor effects—did not significantly differ from the control in any of the treatments (Biden treatment: 1.0 pp decrease, t=−1.00, P=0.320; American treatment: 0.95 pp increase, t=0.91, P=0.362; Trump treatment, 1.2 pp increase, t=1.17, P=0.24).

We observed a null effect, rather than the expected positive descriptive norm effect, in the Biden and American treatments. There are several possible explanations for this. One is that the descriptive social norm did not credibly communicate new information, i.e. most participants already knew the mask-wearing behavior of those who were similar to them, and thus ignored the information that we provided—either because it comported with their existing beliefs or, more likely, that they felt this information was unreliable given their experience. Another possibility is that descriptive norm increased safety concerns and safety-based motives for mask wearing, which canceled out a typical positive norm effect. Other studies during the COVID-19 pandemic found that descriptive norms can alter safety concerns (13). As the focus of this paper was on the impact of outgroup descriptive norm information, we did not investigate ingroup or general norm treatments further.

## Study 2: replication of the Trump norm backfire

Study 2, which was run three months after Study 1, sought to replicate the backfire observed in Study 1’s Trump treatment. We conducted this replication because the backfire effect in Study 1 was not that strongly statistically significant (P=0.027) and such a backfire effect had, to our knowledge, never been previously documented. Thus, we wanted to ensure that the effect was replicable before further investigating the underlying mechanism. Consistent with this, we increased our desired sample size per condition to 500 for this and subsequent studies. Furthermore, during the COVID-19 pandemic, attitudes towards masking—and towards the pandemic in general—were changing relatively quickly (27). Thus, it was also useful to see whether the results of Study 1 were not unique to the particular moment in time we ran that experiment.

### Participants

Study 2 was conducted from 2022 August 15 to 2022 August 24, recruiting Americans using Prolific. Balancing statistical power with available budget, we aimed to recruit 1,000 Democrats who preferred Joe Biden to Donald Trump (500 per condition, allowing more statistical power than Study 1). In total, 1,028 participants (screened by Prolific to be Democrats) began the study; 36 were not allowed to complete the study because they reported preferring Donald Trump to Joe Biden; and 2 participants did not complete the key outcome measure. Thus, our final sample consisted of 990 participants (mean age = 33 years, age range = 18–80; 488 men, 479 women, and 23 who responded “Nonbinary/third gender”).

### Materials and procedure

The procedure was identical to Study 1, except that only the control and Trump treatments were included.

### Results

**Fig. 2.**
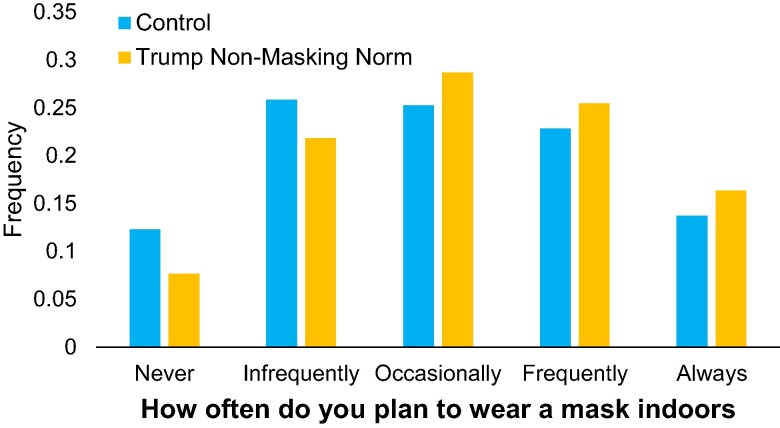
Distribution of masking intentions by condition in Study 2 (N=990). Control participants were given no information; participants in the treatment were told that 9 out of 10 Trump supporters reported not planning to wear masks.

Replicating the results from Study 1, participants in the treatment reported that they intended to wear masks significantly more frequently than participants in the control (d=0.18; t(988)=2.76, P=0.006; Fig. 2). Also replicating the results from Study 1, the treatment caused a marginally significant increase in participants’ expectations of the fraction of Biden supporters wearing masks indoors (2.2 pp, t=1.68, P=0.093) and no significant effect on expectations of the fraction of Trump supporters wearing masks indoors (0.3 pp, t=0.46, P=0.65), likely due to floor effects (as participants in the control expected only 8.2% of Trump supporters to wear masks indoors).

## Study 3: mediation by punishment expectations

Having successfully replicated the social norm backfire in Study 2, we then sought to provide evidence for mechanisms driving this result in Study 3. The first possible mechanism we explored was the one discussed in the introduction: that descriptive norms communicate information about the likelihood of sanctions by one’s ingroup. To do so, we measured the extent to which participants anticipate sanctions from ingroup members for not masking. We also explored a second possible related mechanism: that learning that Trump supporters did not wear masks made mask-wearing a stronger signal of being a Biden supporter—i.e. a stronger signal of ingroup membership (28). To do so, we measured the extent to which participants think that wearing a mask makes a person look like a Biden versus Trump supporter. Finally, we also asked whether the treatment effect varies based on the strength of participants’ identification with the Democratic versus Republican party to assess an identity-based mechanism whereby Biden supporters are simply displaying reactance to the behavior of those they disagree with politically.

### Participants

This study was conducted from 2022 October 21 to 2022 October 26, recruiting Americans using Prolific. As in Study 2, we aimed to recruit 1,000 Democrats who preferred Joe Biden to Donald Trump as president. In total, 1,141 participants (screened by Prolific to be Democrats) began the study; 51 were not allowed to complete the study because they reported preferring Donald Trump to Joe Biden; and 2 participants did not complete the key outcome measure. Thus, our final sample consisted of 1,088 participants (mean age = 35 years, age range = 19–79; 538 men, 512 women, and 38 who responded “Nonbinary/third gender”).

### Materials and procedure

The procedure was identical to Study 2, except that immediately after receiving the control or treatment message and indicating their mask wearing intentions, participants were taken to a screen in which we measured potential mediators. They were asked two questions assessing anticipated ingroup social sanctions for not wearing masks: “How do you think Biden supporters would feel about you not wearing a mask indoors?” and “How do you think your family, friends, and other community members would feel about you not wearing a mask indoors?,” both with 5-point Likert response scale [Not care at all, Slightly negative, Somewhat negative, Negative, Extremely negative]. A third question assessed the signaling value of mask-wearing: “To what extent do you think that wearing a mask indoors makes a person look like a Biden supporter versus a Trump supporter? Wearing a mask indoors makes a person look more like…” [Strongly Trump, Somewhat Trump, Slightly Trump, No effect, Slightly Biden, Somewhat Biden, Strongly Biden]. The order of the three questions was randomized. We also note that, as in Studies 1 and 2, partizanship was measured pretreatment using a 7-point Likert scale from Strongly Democrat to Strongly Republican.

### Results

**Fig. 3.**
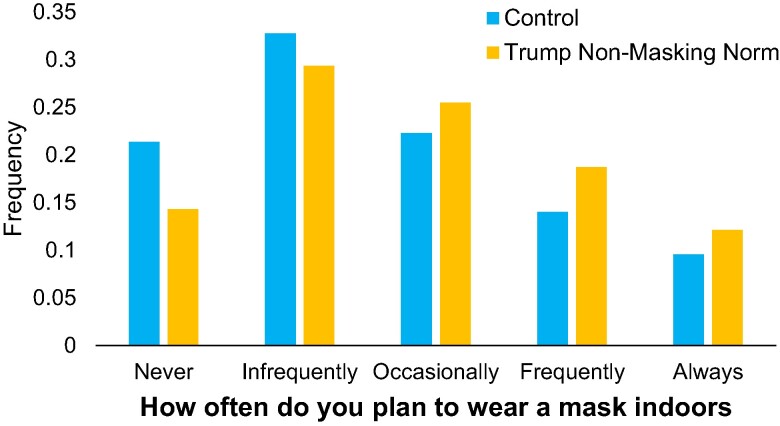
Distribution of masking intentions by condition in Study 3 (N=1,088). Control participants were given no information; participants in the treatment were told that 9 out of 10 Trump supporters reported not planning to wear masks.

Mask wearing intentions by condition are shown in Fig. 3. Replicating the results of Studies 1 and 2, participants in the treatment reported that they intended to wear masks significantly more frequently than participants in the control (d=0.22; t(1,086)=3.65, P=0.0003).

**Fig. 4.**
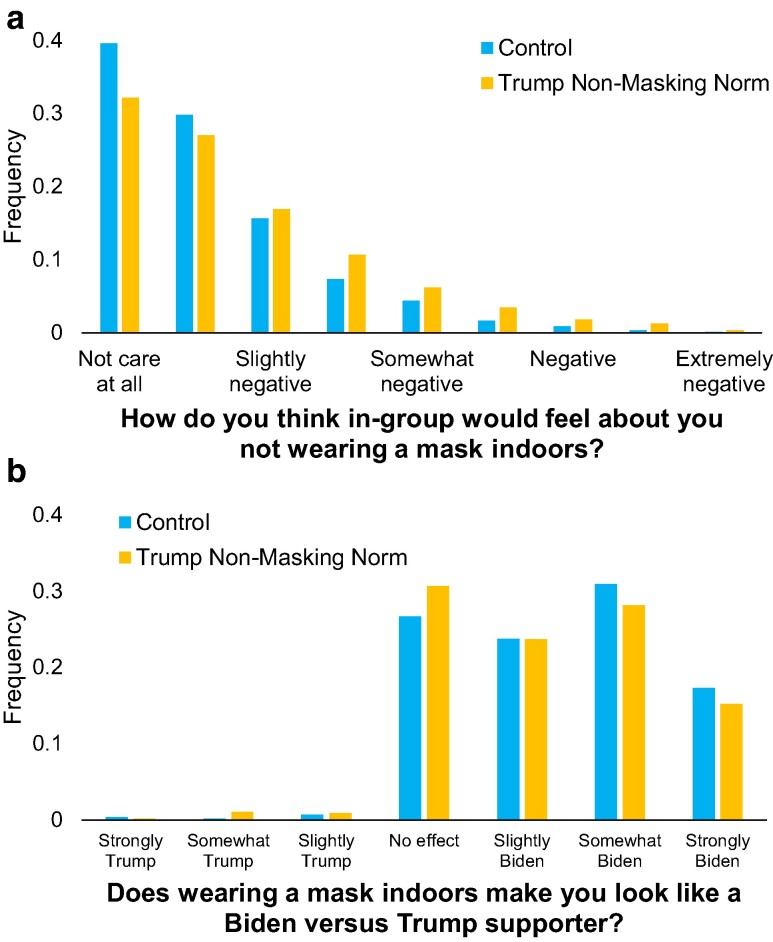
Distribution of responses to the mediators in Study 3 (N=1,088). Control participants were given no information; participants in the treatment were told that 9 out of 10 Trump supporters reported not planning to wear masks. a) Averaged responses to questions about anticipated attitudes towards nonmasking of Biden supporters and participants' family, friends, and community members. b) Perceptions of how wearing a mask affects the extent to which one is seen as Trump versus Biden supporter.

Next, we shed light on the mechanism underlying this effect. Consistent with the sanctioning mechanism, whereby learning that the outgroup engages in a given behavior communicates that the ingroup sanctions this behavior, we find that the treatment significantly increased participants’ perception that not wearing masks indoors would be seen negatively by Biden supporters (d=0.22; t(1,085)=3.69, P=0.0002) and by their friends, family and community (d=0.21; t(1,085)=3.43, P=0.0006); see Fig. 4a. To contextualize these effect sizes, we note that when averaging the two items together, we find that 30.6% of participants in the control think ingroup members will see nonmasking at least slightly negatively, compared with 40.8% in the treatment—a 33.4% increase.

**Fig. 5.**
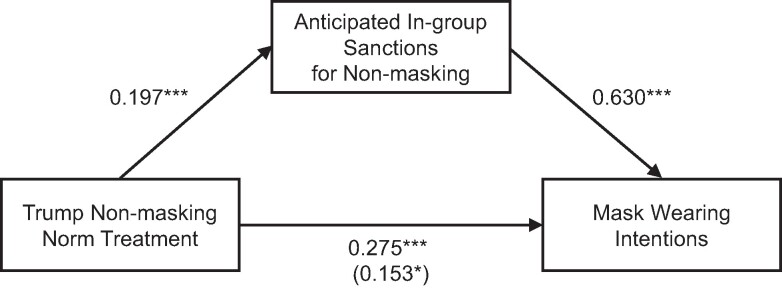
Regression coefficients for the effect of the Trump nonmasking descriptive norm treatment on mask wearing intentions, as mediated by the perceived ingroup norm around mask wearing (i.e. the perception that Biden supporters and friends/family will negatively judge not wearing a mask). The effect of the Trump norm treatment on mask wearing controlling for the perceived ingroup norm is shown in parentheses. *P<0.05, ***P<0.001.

Furthermore, we find that 44.4% of the treatment effect on mask wearing intentions is mediated by an aggregate anticipated social sanctions measure formed by averaging responses to the two anticipated sanctions questions (indirect effect b=0.122, bias-corrected bootstrapped 95% CI [0.066, 0.178]); see Fig. 5. Effects are qualitatively equivalent when using responses to either question individually as the mediator, instead of the aggregate measure.

We also observe that—similarly to Studies 1 and 2—the treatment significantly increased participants’ expectations regarding the percentage of Biden supporters who planned to wear masks in the following month (2.9 pp, t=2.05, P=0.041). However, when controlling for participants’ expectations regarding the percentage of Biden supporters who planned to weak masks, anticipated sanctions continues to mediate the treatment effect on mask wearing intentions (30.1% mediation, indirect effect b=0.061, bias-corrected bootstrapped 95% CI [0.025, 0.096]). Conversely, when controlling for anticipated sanctions, the percentage of Biden supporters who planned to weak masks did not significantly mediate the treatment effect on mask wearing intentions (6.5% mediation, indirect effect b=0.010, bias-corrected bootstrapped 95% CI [−0.040, 0.054]).

In contrast, we do not find support for the signaling mechanism. We find a small and only marginally significant effect of the treatment on participants’ perception that wearing a mask indoors makes a person look like a Biden supporter (d=0.12; t(1,085)=1.93, P=0.053); see Fig. 4b. Furthermore, when conducting a mediation analysis, we find a *negative* indirect effect (b=−0.005, bias-corrected bootstrapped 95% CI [−0.007, −0.003]), such that the effect of the treatment was slightly *larger* when controlling for perceptions of mask wearing making a person look like a Biden supporter.

We also do not find evidence for an identity-based mechanism, whereby Biden supporters who identify more strongly as Democrats are more repelled by the Trump supporters and thus more disinclined to act like them: the treatment effect was not significantly moderated by partizanship (b=0.074, t=1.23, P=0.218 without controls; b=0.091, t=1.41, P=0.158 when controlling for age, gender, income, education, and race, as well as their interactions with the treatment). Furthermore, the (nonsignificant) partizan moderation was directionally opposite from what an identity-based mechanism would predict, as the treatment effect was directionally larger for participants who were more right-leaning.

## Study 4: a nonpolarized context

Study 3 provided evidence that in the context of a highly polarized behavior—mask wearing—learning that outgroup members engaged in a given behavior increased expectations that ingroup members would sanction that behavior. In Study 4, we test a related theoretical prediction: in nonpolarized contexts (i.e. contexts where both groups could be expected to hold similar norms), learning that outgroup members engage in a behavior should work as usual, increasing—rather than decreasing—behavioral intentions. This is because, in a nonpolarized context, outgroup members and ingroup members are generally expected to abide by the same social norms. Thus, outgroup members’ behavior can be informative about how one is expected to behave by—and the sanctions one should anticipate receiving from—ingroup members (so long as ingroup and outgroup norms are perceived as sufficiently similar (2, 19, 21)).

To test this theoretical prediction, we switch to a nonpolarized domain: dishonesty (specifically, lying for personal gain in a coin-flipping task), a behavior which is widely seen as unethical. We use the target groups as studies 1–3 (Trump and Biden supporters).

### Participants

Study 4 was conducted from 2022 November 14 to 2022 November 25, recruiting Americans using Prolific. As in Studies 2 and 3, we aimed to recruit 1,000 participants. Unlike Studies 1–3, in this study we did not restrict participation based on preference for Biden over Trump. In total, 1,106 participants began the study; eight participants did not complete the key outcome measure. Thus, our final sample consisted of 1,098 participants (mean age = 36 years, age range = 18–80; 538 men, 531 women, and 29 who responded “Nonbinary/third gender”).

### Materials and procedure

The study began with a consent form, followed by a series of demographic questions, including “Who would you rather have as President?” (Joe Biden/Donald Trump). After completing the demographic questions, participants were randomized into the control or treatment. In the control, participants received the following instructions:

In this part of the survey, you may be able to earn additional money. We will ask you to flip a virtual coin using this coin-flipping website. If the coin lands on heads, you get an extra 30 cents. If the coin lands on tails, you get no additional money. In a recent study, we asked other people to do this coin flip task. Now it is your turn to flip a coin.

**Important: Please do not cheat! Actually flip the virtual coin, and report the result of the very first flip.**

What did it land on?

In the treatment, the instructions were identical except that following the line “In a recent study, we asked other people to do this coin flip task”, participants who reported a preference for Biden were told “Out of 10 Trump supporters we asked, 9 said that their coin flip came out ‘Heads’.” while participants who reported a preference for Trump were told “Out of 10 Biden supporters we asked, 9 said that their coin flip came out ‘Heads’.”

In both conditions, when participants clicked on the link, they were taken to a coin flipping web app created using https://scratch.mit.edu, in which the first flip always came out tails, while all subsequent flips were random. Thus, if participants followed the directions and did not cheat, they should all have reported tails. (Participants were informed about this deception following the conclusion of the study, and all participants were paid the 30 cent bonus.)

They were then asked how their friends, family, and other community members would feel about them lying in the coin-flipping task, and then asked what fraction of Biden and Trump supporters they thought were going to say they got heads. Finally, participants in the treatment conditions received a debriefing stating “Earlier in the study, we told you 9 out of 10 people from a recent study said they got heads on their coin flip. However, the choices of those particular 10 people do not reflect the choices of all of the participants as a whole.”

### Results

For comparability to the previous studies, we primarily focus on the N=642 participants who preferred Biden to Trump. We note that 1 (0.2%) of these participants did not answer the community perceptions question, 19 (3.0%) did not enter an expectation for Trump supporters, and 28 (4.4%) did not enter an expectation for Biden supporters, as these questions did not require a response (unlike the main coin flip question).

In contrast to Studies 1,2, and 3, we did not observe a descriptive norm backfire, but instead observed a standard descriptive norms effect (in which participants adopted the described behavior): informing Biden supporters that most Trump supporters were reporting “heads” in the coin task lead to significantly more Biden supporters themselves reporting “heads” in the treatment (19.6%) compared to the control (13.3%; $\chi^{2}(1)=4.68$, P=0.031). That is, in this nonpolarized context, providing information about the behavior of outgroup members caused ingroup members to adopt that same behavior.

Also in contrast to Studies 1, 2, and 3, the treatment significantly increased the fraction of Trump supporters who Biden supporters expected to report “heads” (67.8% in control, 75.4% in treatment; t(621)=4.72, P<0.001). The effect on the fraction of Biden supporters they expected to report “heads” was small and not significant (53.2% in control, 54.4% in treatment; t(612)=0.74, P=0.46), as was the effect on their expectations of how negatively their family, friends, and community members would view lying on the coin task (M=2.11 in control, M=2.20 in treatment, t(639)=0.98, P=0.329).

Interestingly, Biden supporters largely anticipated that most participants from both parties would be honest (in control, modal anticipated fraction of both Biden and Trump supporters reporting “heads” = 50%). Furthermore, Biden supporters’ expectations of differences in honesty between Biden and Trump supporters were much smaller for the coin task (e.g. median difference in predicted percent of Biden versus Trump supporters reporting “heads” was only 8 percentage points) compared to masking (e.g. in Study 1, where median difference in predicted percent of Biden versus Trump supporters wearing masks was 39 percentage points). These observations support our expectation that the coin task is a substantially less polarized context than masking, and that Biden supporters perceived honesty as largely normative for both ingroup and outgroup members.

Finally, we note that among the 41.7% of participants who preferred Trump to Biden, an equivalent fraction reported “heads” in the treatment (20.3%) and control (20.9%; chi2(1) = 0.03, P=0.865)—despite the treatment successfully increasing expectations regarding the fraction of Biden supporters reporting “heads” (61.6% in control, 64.9% in treatment; t(447)=3.15, P=0.002). Thus, the descriptive norms treatment did not have the typically observed effect among Trump supporters (although also did not backfire).

## Discussion

Across three studies, we present robust evidence of a descriptive social norm backfire: informing Biden supporters that Trump supporters were not wearing masks significantly increased their mask-wearing intentions. We also provide evidence consistent with this treatment effect being driven by heightened concern that not wearing a mask would be seen negatively by friends, family, and other Biden supporters. In a fourth study, we provide evidence that these backfire effects are unique to polarized contexts by showing that the effect does not extend to the nonpolarized behavior of dishonesty in a coin-flipping task.

Our results contribute to theorizing about the mechanism through which descriptive social norms operate, helping to tie the “behavioral” literature on descriptive social norms when used as nudges to the “functional” literature that explores the role of social norms in supporting human cooperation (1, 18, 20, 25, 29–31). Our findings support an account whereby descriptive social norms change behavior by changing people’s perceptions of whether that behavior is acceptable or prohibited—and will thus be rewarded or punished—within their social group. It has been suggested that people only respond to information about those in their reference group, because the behavior of those outside the reference group does not provide information for drawing inferences about relevant norms in one’s own group (2, 18, 20). Here, we extend this theory by positing that for highly polarized contexts (like masking), behavior of people outside of the reference network can still have an impact—if those people are part of the outgroup. In this case, learning that out-party members engaged in a behavior provides information that that behavior is likely to be frowned upon by the ingroup—leading to avoidance of the behavior. Importantly, this norm-driven “backfire” effect does not involve any direct instruction to behave in a manner consistent with the outgroup, and thus is distinct from reactance effects in which people respond negatively to feeling that they are being coerced (32).

From a practical perspective, our results are striking given how hard changing intentions to engage in COVID-19 prevention behaviors has proved to be (22, 33–37). This is particularly true given how late into the pandemic our studies were conducted, and thus what a large volume of masking-related messaging participants had already been exposed to (e.g. from government public health initiatives). The ability of our treatment to increase mask-wearing intentions further demonstrates the power of social norms for shaping behavior. Conversely, however, we also fail to observe the expected descriptive social norm (nonbackfire) effect in various settings—e.g. informing Biden supporters about Americans or other Biden supporters not planning to mask in Study 1, and informing Trump supporters about Biden supporters cheating in Study 4. These null results highlight the practical lesson that descriptive social norm interventions may not always yield the expected results.

Our focus on outgroup nonmasking norm information is complementary with work earlier in the COVID-19 pandemic before masks became overly politicized, which found that general descriptive norm information that others (10) or Americans (12) were wearing masks increased mask-wearing intentions, and that an outgroup descriptive norm treatment (56% of Republicans planning to wear masks) had an effect that, while not statisticially distinguishable from zero or the effect of an ingroup descriptive norm, was slightly larger than the effect of the ingroup descriptive norm (12). The effect of providing information about Americans was smaller in our Study 1 than in these previous studies, perhaps due to limitations in the credibility of our treatment as already discussed, and perhaps because these previous studies were conducted sufficiently early in the pandemic that people’s perceptions of others’ masking were still relatively malleable. The effect of our outgroup descriptive norm on masking intentions was somewhat larger than that reported by Carey et al. (12). This is perhaps because we told participants that 9 out 10 Trump supporters would wear masks, whereas in the descriptive norm employed by Carey et al., the share was just 56%; and, also, perhaps because we referenced Trump supporters rather than Republicans writ-large, and thus accentuated the polarization of the outgroup. However, there were many other differences between the studies, and, as previously noted, perceptions of others’ masking and attitudes towards masking were changing rapidly during this period.

Of course, our studies have various limitations. First, Studies 1 to 3 occurred during a period when norms towards masking were in flux, and are unlikely to generalize to attitudes towards masking in another time or political environment. It would be valuable to document additional examples of descriptive norms backfiring for highly polarized behaviors. We also caution against using the results herein to predict current or future attitudes towards masking. Second, we emphasize that we measure masking intentions, rather than actual masking behavior, and it is possible that actual mask-wearing behavior would not be as sensitive as our measures of intentions might suggest; intentions have been shown to be correlated with a variety of real-world behaviors (38), albeit, not specifically masking. Third, in looking for evidence illuminating the mechanism by asking participants for their perceptions of anticipated sanctions and the signaling value of masks, we are reliant on participants conscious consideration of these factors. Critically, if norms are internalized (1), these explicit reports are likely to understate the importance of such mechanisms (indeed, many participants reported that ingroup members do not care about them not wearing masks, which might be because the effect of the sanctioning mechanism is, at least for some participants, internalized, and they are not conscious of it). Thus, while we do not find evidence consistent with the signaling mechanism, it might still be present, and people might just not be consciously aware of the descriptive norm information’s effect on their perceptions. Third, although we believe Study 4 is helpful for highlighting the importance of the hyper-partizan context in driving our backfire effect, there are many differences between Study 4 and Studies 1–3 beyond simply the level of polarization of the task. For instance, whereas when many others are not masking, the individual benefits to masking increase, there is no such effect on lying: the individual material costs or benefits of lying are independent of others’ behavior. Furthermore, Study 4 involved an actual incentivized decision, whereas Studies 1-3 relied on behavioral intentions. Such differences could also contribute to why the descriptive norm worked differently in Study 4 than in the previous three studies. Fourth, we did not use nationally representative samples. Thus, it is hard to know how to interpret the partizan differences we observed in Study 4 (where the outgroup descriptive norm had no effect on Trump supporters). Future work should replicate our experiment using a more representative sample.

Finally, we conclude by reflecting on the relevance of our findings in light of concerns about widespread media coverage—and social media denunciations—of people flouting COVID-19 recommendations such as masking and social distancing. It could very reasonably have been expected that such coverage would reduce compliance via descriptive social norm effects. The data presented here suggest that rather than reducing compliance across the board, such coverage may have instead been polarizing—reducing compliance among in-party members (e.g. those on the right), but increasing compliance among out-party members (e.g. those on the left) and helping to link compliance to group identity (37).

## Data Availability

Materials, data, and code for all experiments is available at https://osf.io/82fh7/
